# The Outbreak of Influenza

**Published:** 1920-05-22

**Authors:** 


### ST. KILDA.

### The Outbreak of Influenza.

The provision of medical service for the inhabitants of St. Kilda is a problem by itself. During the war, when the island was in touch with the mainland by wireless and within the beat of Naval patrols, the Highlands and Islands Medical Service Board had no great difficulty in communicating with the nurse maintained by them on the island or in responding at once to urgent calls for assistance. It was possible, for example, with the ready co-operation of the naval authorities and the Medical Officer in Stornoway, to have a doctor in attendance on a difficult confinement case in St. Kilda in a remarkably short time after the wireless message for medical aid was sent out. But with the withdrawal of the patrols and the discontinuance of the wireless station on St. Kilda, the Scottish Board of Health, who now administer the Highlands and Islands Medical Service Fund, are faced with difficulties which will be clear from a statement of the facts, so far as they are known at present, connected with the recent outbreak of influenza among the residents.

On April 16 Nurse Mackenzie, the Board's nurse on St. Kilda, wrote her message intimating the outbreak and asking for stores. Her message was taken to Fleetwood by a trawler and telegraphed to the Board, reaching them on April 26. At the Board's request the General Post Office in Edinburgh immediately made inquiries to find out when a boat was likely to leave any of the usual ports for St. Kilda, and learned that a trawler was expected to sail from Fleetwood on the morning of April 28. An order for the necessary medicines and instructions to the nurse were telegraphed to Fleetwood to go by this trawler, but the postmaster there intimated that the sailing had been cancelled and that no other boat was expected to leave the port for the vicinity of St. Kilda for a time. Nothing more could be done till the Post Office informed the Board of Health on April 30 that word had been received from Aberdeen that the trawler *Active*, which had brought back later news of conditions on the island, was returning to St. Kilda on Sunday, May 2. In view of the serious nature and extent of the outbreak as reported by Skipper D. Craig, D.S.C., of the *Active*, Dr. Shearer, one of the Board's Medical Officers, left Edinburgh on the Saturday to join the boat; but, although the sailing was fixed for the 2nd, weather conditions delayed the trawler's departure from Aberdeen till Monday, the 3rd instant. If, as is probable, the *Active* reached St. Kilda late on the 4th, the time between the date of Nurse Mackenzie's first message and Dr. Shearer's arrival on the island is not far short of three weeks.

The foregoing *résumé* of the steps taken to meet the situation will indicate the difficulties to be contended with in looking after the physical well-being of a remote community, the members of which seem to be peculiarly susceptible to infections of certain types. It is fortunate that neither the Highlands and Islands Medical Service Board nor the Scottish Board of Health have had difficulty in finding highly trained and capable nurses willing to undertake the responsibility of serving in this lonely post.

